# Plant Metabolomics in the Global Scenario of Food Security: A Systems-Biology Approach for Sustainable Crop Production

**DOI:** 10.3390/ijms19072094

**Published:** 2018-07-19

**Authors:** Marcello Iriti, Sara Vitalini

**Affiliations:** 1 Department of Agricultural and Environmental Sciences, Milan State University, via G. Celoria 2, 20133 Milan, Italy; sara.vitalini@unimi.it; 2 National Interuniversity Consortium of Materials Science and Technology, via G. Giusti 9, 50121 Firenze, Italy

In the past few years, global food security progressively worsened, particularly in sub-Saharan Africa and some parts of southeastern and western Asia. This was mainly caused by the increased number of violent conflicts around the world, especially in developing countries. The situation is often exacerbated by adverse climate-related events, such as droughts or flooding. According to the most recent estimates by the Food and Agricultural Organization (FAO), world hunger and undernutrition appear to be on the rise again. Indeed, the global prevalence of undernourishment decreased from 14.7% (900 million people) in 2000 to 10.8% (775 million people) in 2013, while, in 2014 and 2015, this trend came to a halt (10.7% and 10.6%, respectively). Finally, in 2016, the number of undernourished people increased to 815 million (11%), up from 777 million in 2015, reaching the levels registered in 2012 (Figure 1 [1]). Civil conflicts, climate change, and crop production are deeply intertwined [2]. In particular, global warming poses a threat to food security. At the same time, the effects of conflicts on food security are dire, while the world’s population could increase to 9.7 billion people by 2050, compared to today’s 7.5 billion.

In this context, the large-scale study of the total small-molecule array of a plant biological system can represent a powerful tool to understand the plant’s physiological status under normal and stressful conditions. In their environment, plants experience abiotic and biotic stresses. Therefore, plants produce a wide variety of secondary metabolites (small molecules ≤10 kDa) to cope with pathogenic microorganisms, phytophages, competing plants, environmental pollutants, and adverse climatic changes. Noteworthy, phytochemicals not only defend plants against their enemies, but also secure their growth, development, and reproductive success, with flower pigments and volatile compounds attracting pollinators. The metabolome is, thus, a central pillar in plant systems biology, integrating all upstream “-omics” (genomic, transcriptomic, and proteomic), and providing the molecular phenotype of a biological system [3].

Due to being a core trait, contributions of metabolomics to basic and applied plant sciences will be very promising in the near future, as reported in diverse fields (Table 1). Various abiotic stresses were investigated. Sulfur treatment alleviated aluminum toxicity in citrus (*Citrus grandis*) plants by activating a series of metabolic mechanisms responsible for tolerance, with a high impact in the field of metal toxicity in crop plants [4]. The arctic plant, *Dracocephalum palmatum* (used as a medicinal and food plant), exhibited a dramatic change in its phytochemical profile at low temperatures, mainly concerning lipophilic metabolites eco-physiologically involved in cold acclimation, pointing towards a low-temperature cultivation of useful arctic plants for bioactive enrichment [5]. Lipidome changes were recorded in thyme (*Thymus* spp.) plants tolerant or sensitive to water deficiency under severe drought conditions, thus elucidating the mechanisms of plant adaptation and tolerance to water deficit [6]. In the field of plant fitness and crop yield, the developmental shift in primary and secondary metabolism in cucumber (*Cucumis sativus*) fruit was investigated, providing potential applications in quality improvement of cucumber fruit [7]. In addition, analysis of the seed-coat composition of six pea (*Pisum* spp.) genotypes possessing different propensity to dormancy/germination showed higher levels of hydroxylated long-chain fatty acids in dormant genotypes [8]. Similarly, symbiotic interactions represent another relevant issue. Bean (*Phaseoulus vulgaris*) root nodules inoculated with a mutant strain of the rhizobium, *Paraburkholderia phymatum*, revealed significantly higher levels of flavonoids, a class of phytoalexins [9]. The topic of plant resistance to pests was also studied. In wheat (*Triticum aestivum*) cultivars, variation in the composition of cuticular waxes associated with resistance to insect pests was demonstrated, thus supporting the potential of breeding for an important phenotypic trait [10]. Finally, in the field of health benefits of plant products, a new analytical method was developed for ginsenoside profiling and quality control of ginseng (*Panax ginseng*) roots, one of the most important herbal products, at different ages in herbal markets [11]. At the end of this brief description, it seems that most studies focused on the eco-physiological aspects of plant resilience to detrimental environmental conditions, as emphasized in a seminal review article touching on most of these issues [12], with the non-polar metabolome (lipidome) mainly involved in the plant’s response to abiotic/biotic stresses.

Therefore, focusing on the practical applications of metabolomics, it can really be a useful approach to improve both crop yield and quality, thus contributing to reaching the goal of modern agriculture, i.e., to feed the entire world in a sustainable way.

## Figures and Tables

**Figure 1:**
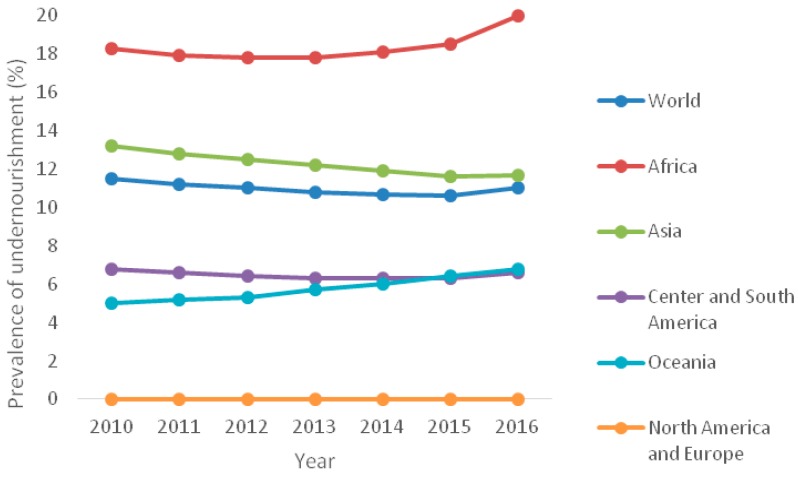
The prevalence (%) of undernourishment in the world by region, 2010–2016 (source: Food and Agricultural Organization, FAO [1]).

**Table 1:** Contribution of metabolomics to basic and applied plant sciences.

Topic	References
**Abiotic stresses**	
Metal alleviation in *Citrus*	[4]
Eco-physiological mechanisms of cold adaptation of arctic plants	[5]
Drought stress in aromatic plants	[6]
**Plant fitness/crop yield**	
Regulation of fruit development in cucurbits	[7]
Mechanisms of seed dormancy in legumes	[8]
**Symbioses**	
Nitrogen-fixing symbiosis in legume root nodules	[9]
**Plant resistance to pests**	
Composition of cuticular waxes in cereals	[10]
**Human health**	
Profiling of ginsenosides in *Panax ginseng*	[11]
**Plant ecology**	[12]
